# Supplementary material for: Network integration of multi-tumour omics data suggests novel targeting strategies
Source: Nat Commun. 2018 Oct 30;9:4514. doi: 10.1038/s41467-018-06992-7 (PMC6207774; doi:10.1038/s41467-018-06992-7)
Supplement: Supplementary file 1 — Supplementary Information [file 41467_2018_6992_MOESM1_ESM.pdf]

Supplementary Information

Network integration of multi-tumor omics data suggests novel targeting strategies

Valle IF, Menichetti G, Simonetti G, *et al*.

Supplementary Note 1

In this study, we analyzed the gene expression of 2378 samples from 11 tumor types in order to define multi-tumor gene signatures (Supplementary Table 1). First, we created a backbone network that we refer as BioPlex-Ontocancro network. This network was built from cancer-related genes (as defined in the Ontocancro database) that presented protein interaction profiles in the BioPlex Network (http://bioplex.hms.harvard.edu/). This network was composed by 760 nodes (of which 511 nodes were not isolated) and 981 edges (Supplementary Table 2, Supplementary Figure 1). After defining clusters of tumors (see Methods in main text), we combined the BioPlex-Ontocancro with the correlation matrices derived from gene expression profiles of all patients in each multi-tumour cluster. The final networks (Supplementary Table 2) are shown in Supplementary Figures 2, 3 and 4, in which two genes present a link if their respective proteins physically interact and if their gene expression correlate significantly among cluster patients. The network context of the cluster signatures 1, 2, and are shown in the Figure 1 (Main Text), Supplementary Figure 5, and Supplementary Figure 6, respectively. The Supplementary Table 3 shows the comparison different centrality measures (Spectral, Betweenness, and Strength) in the cluster networks.

Supplementary Table 1 – TCGA datasets. List of tumours and their respective number of gene expression arrays

Abbreviation	Cancer	Number of patients
BRCA	Breast invasive carcinoma	593
COAD	Colon adenocarcinoma	172
GBM	Glioblastoma multiforme	595
KIRC	Kidney renal clear cell carcinoma	72
KIRP	Kidney renal papillary carcinoma	16
LGG	Brain lower grade glioma	27
LUAD	Lung adenocarcinoma	32
LUSC	Lung squamous cell carcinoma	155
OV	Ovarian serous cystadenocarcinoma	590
READ	Rectum adenocarcinoma	72
UCEC	Uterine corpus endometrial carcinoma	54
	Total	2378

Supplementary Table 2 – Network Properties. The table shows the main topological features of the cluster networks. Cluster 1: COAD and READ; Cluster 2: LUAD, LUSC, GBM, OV, BRCA, and UCEC; and Cluster 3: LGG, KIRC and KIRP.

	BioPlex-Ontocancro	Cluster 1	Cluster 2	Cluster 3
Clustering Coefficient	0.25	0.21	0.19	0.18
Connected Components	24	41	42	41
Network Diameter	16	18	19	18
Avg Path Length	6.52	7.41	6.88	7.31
Avg Degree	3.84	3.2	3.14	2.98
Number of Nodes	511	406	408	410
Number of Edges	981	650	642	612

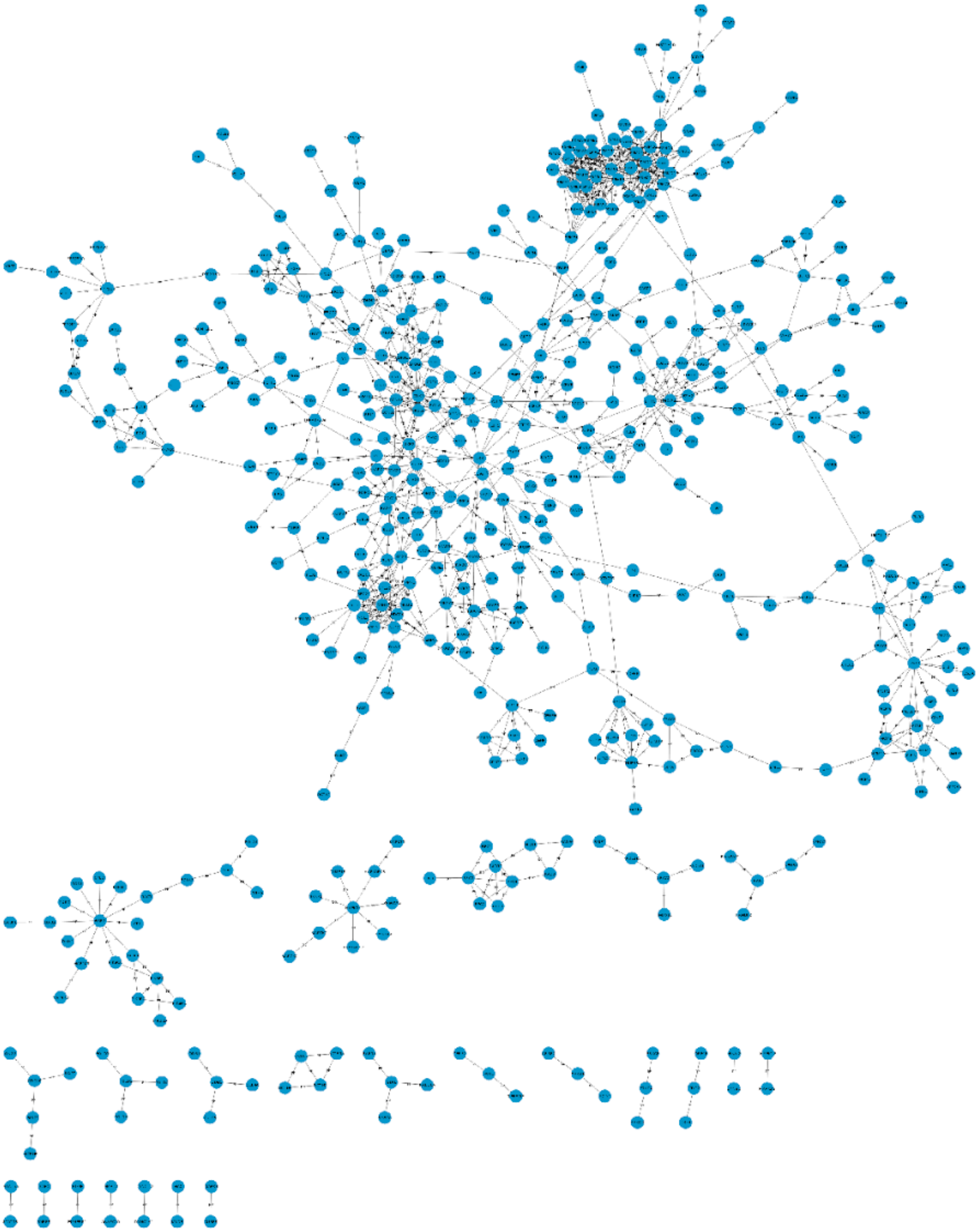

Supplementary Figure 1 – Bioplex-Ontocancro Network. Network built from the 760 genes found in both BioPlex protein-protein interaction network and Ontocancro database (511 connected and 249 isolated nodes)

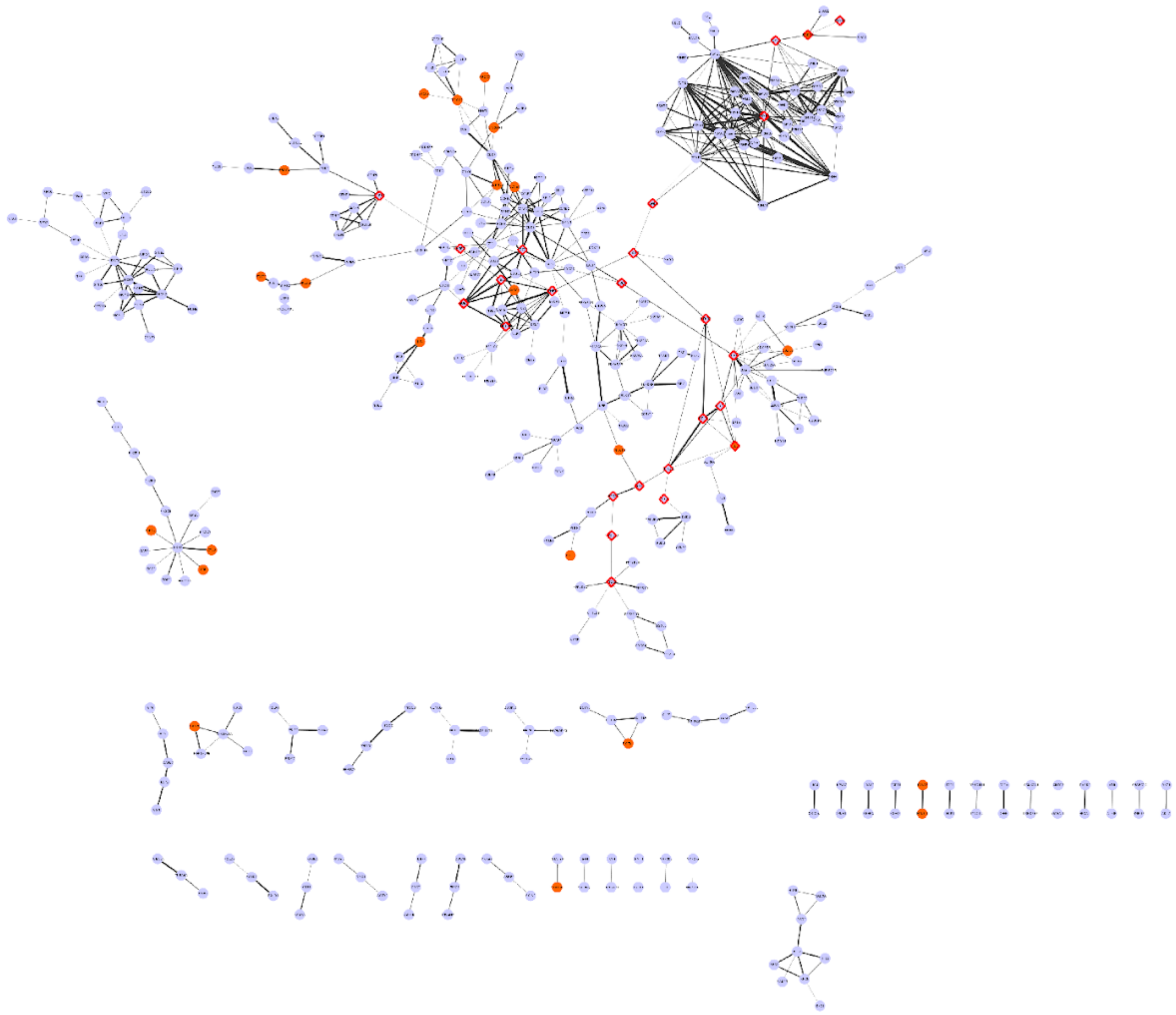

Supplementary Figure 2 – Cluster 1 network. Diamonds with red borders: cluster 1 signature; orange circles: mutated genes. Protein interactions not present in the largest component are presented in the Supplementary Table 16.

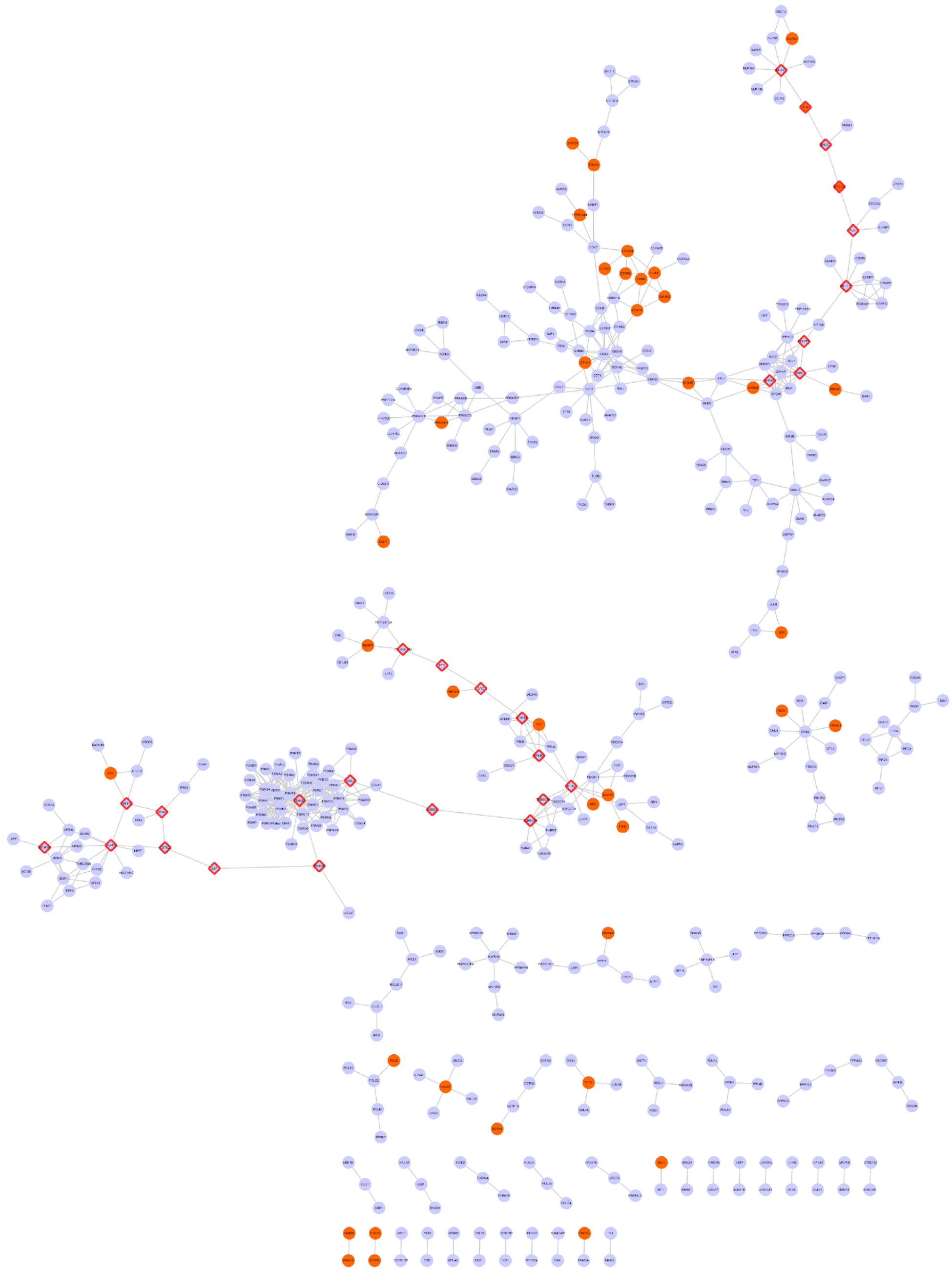

Supplementary Figure 3 – Cluster 2 network. Diamonds with red borders: cluster 2 signature; orange circles: mutated genes Protein interactions not present in the two largest components are presented in the Supplementary Table 17.

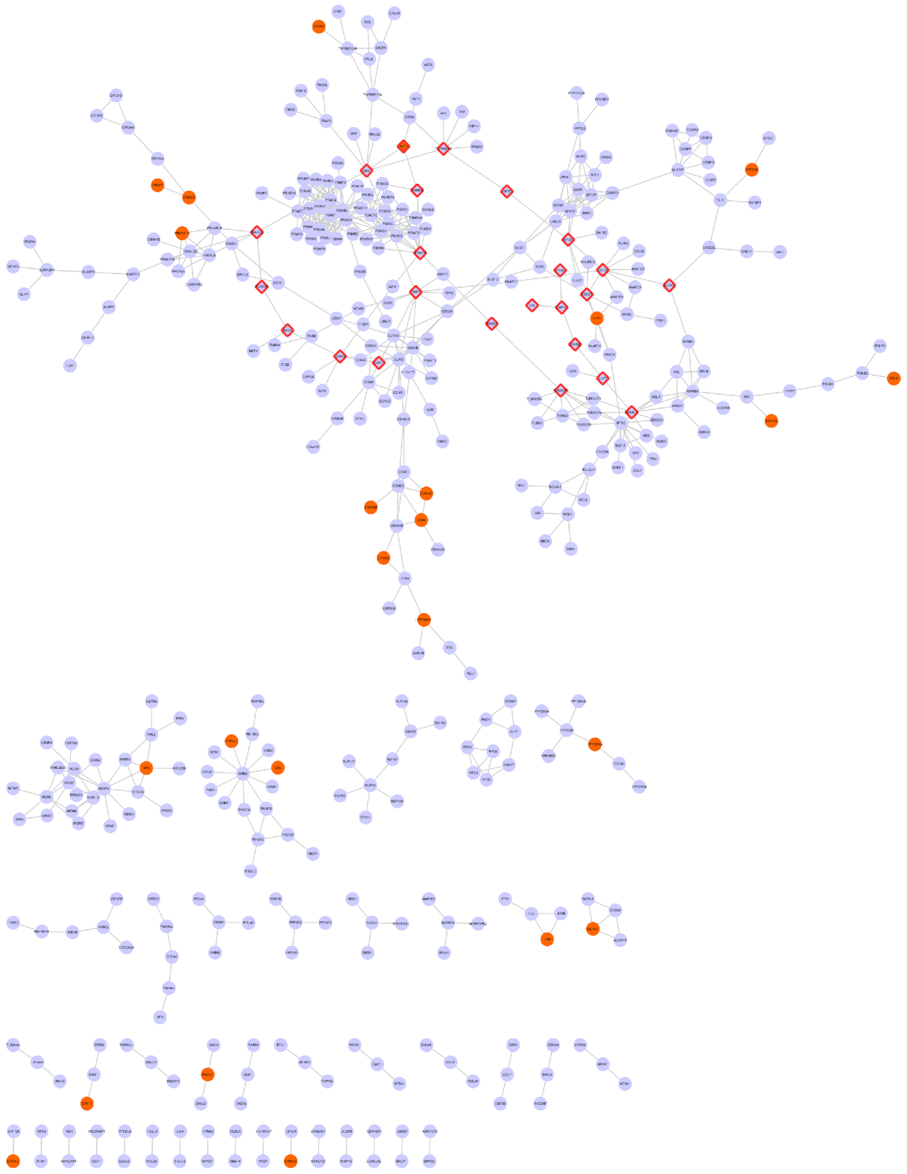

Supplementary Figure 4 – Cluster 3 network. Diamonds with red borders: cluster 3 signature; orange circles: mutated genes. Protein interactions not present in the largest component are presented in the Supplementary Table 18.

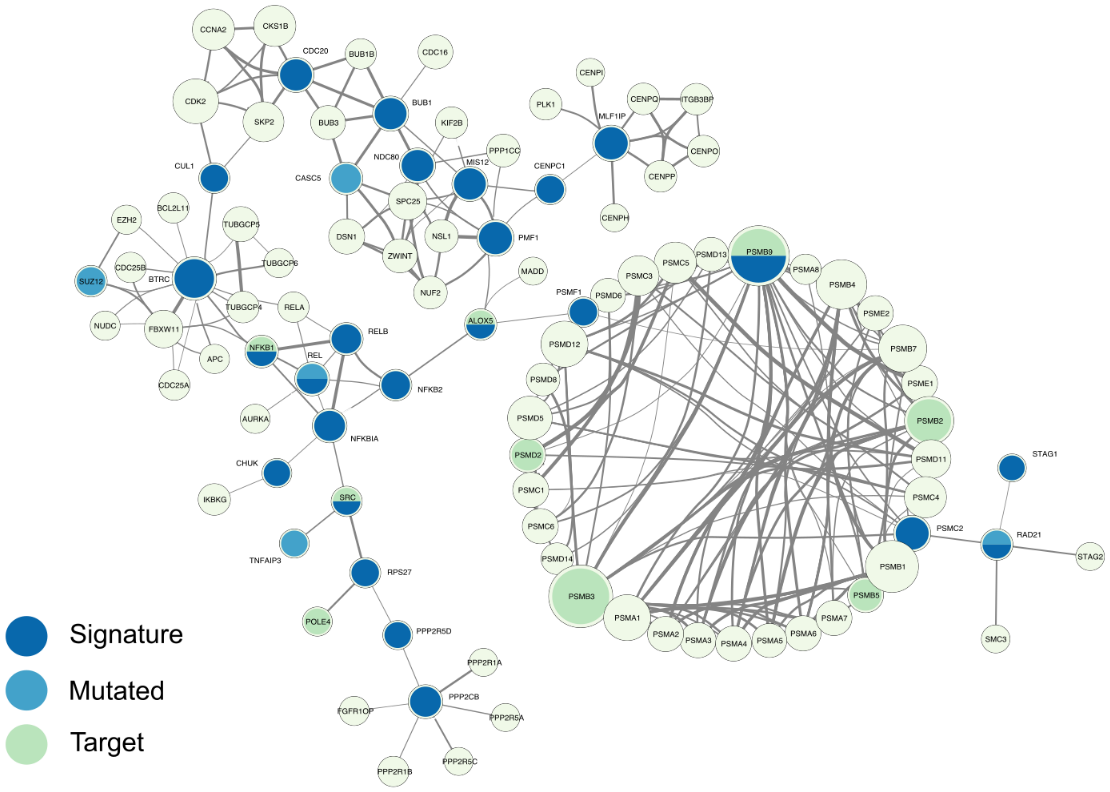

Supplementary Figure 5 – Network context of cluster 1 signature genes. Network composed by the first neighbors of cluster 1 signature genes in the BioPlex-Ontocanco network. Node sizes are proportional to their degree in the network and edge thickness is proportional to the normalized (CLR) co-expression between genes.

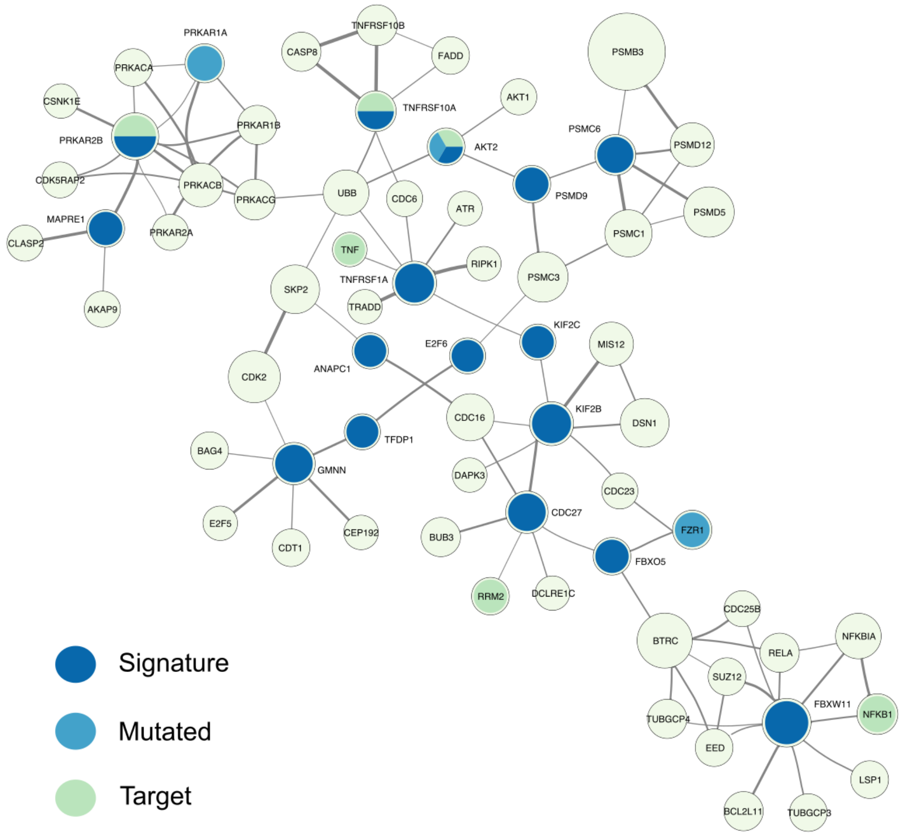

Supplementary Figure 6 - Network context of cluster 3 signature genes. Network composed by the first neighbors of cluster 3 signature genes in the BioPlex-Ontocanco. Network. Node sizes are proportional to their degree in the network and edge thickness is proportional to the normalized (CLR) co-expression between genes.

Supplementary Table 3 - Spearman's rank correlation values for the node centrality measures (Spectral Centrality SC, Betweenness Centrality BC, strength W) of the 3 clusters. The results refer to the whole node list ("All") or only to the signatures ("Sign"). We remark the drop in correlation when considering only the signature genes.

	**Cluster 1**	**Cluster 2**	**Cluster 3**
**All: SC vs BC**	0.77	0.66	0.65
**All: SC vs W**	0.39	0.36	0.23
**Sign: SC vs BC**	0.40	0.42	0.08
**Sign: SC vs W**	-0.08	-0.37	-0.008

Supplementary Note 2

The Context Likelihood of Relatedness (CLR) algorithm, used in our study to filter spurious and false correlations, removed about 55% of the initial 760x760 correlation matrix values by imposing z>0 (see Methods). In this Supplementary Section, we applied the algorithm with increasing levels of stringency (Supplementary Figure 7).

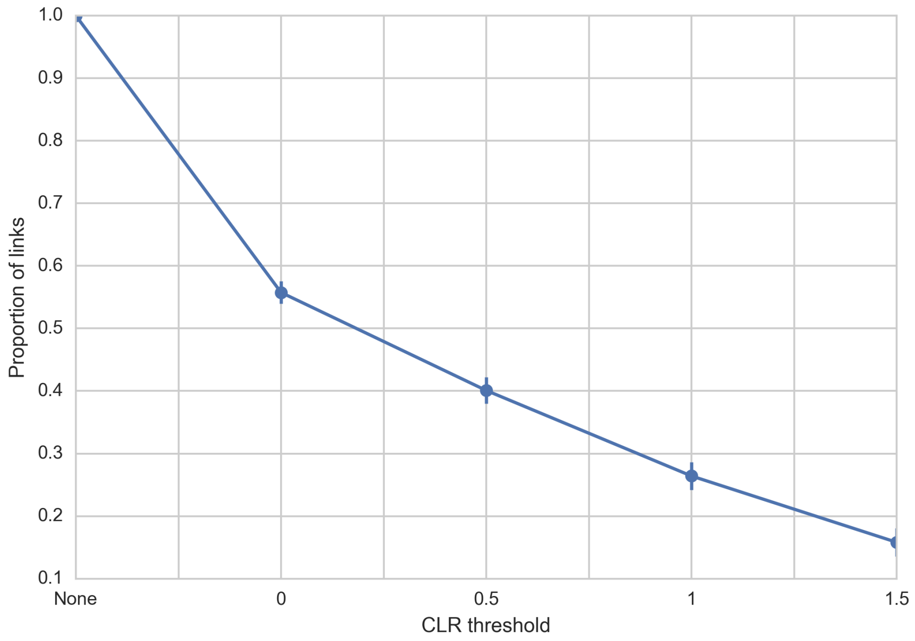

Supplementary Figure 7 - Proportion of significant links selected from the original PPI through increasing CLR thresholds.

Even with increasing thresholds, some subgroups of tumors tended to cluster together (except for the highest threshold z>1.5, in which only 15% of the original link are selected). Examples are: COAD and READ; KIRC and KIRP; and the subgroup LUSC, GBM and OV (Supplementary Figure 8).

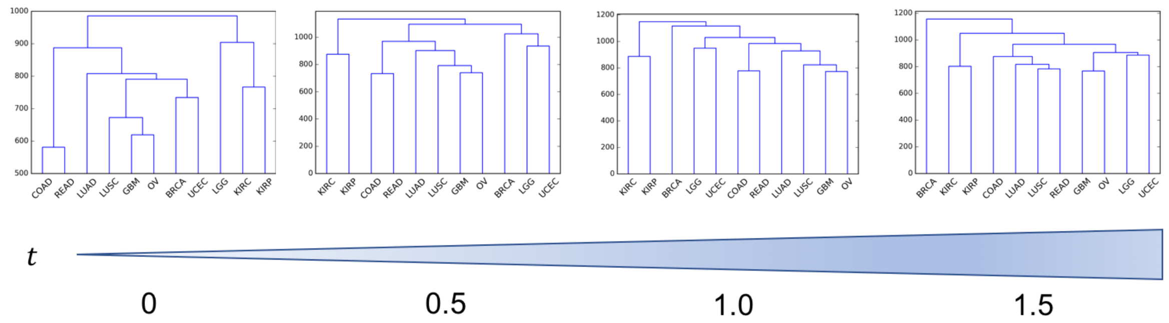

Supplementary Figure 8 - Clustering outcomes for different CLR threshold values

Supplementary Note 3

We show an example of how of combining gene expression correlation and Bioplex-Ontocancro PPI networks changes the resulting PPI network. Considering the gene BTRC have 16 interacting proteins in the initial BioPlex-Ontocancro network. By removing links due to non-significant correlation values (by CLR filtering), BTRC results in 14 and 11 interactions in clusters 1 and 2, respectively (Supplementary Figure 9).

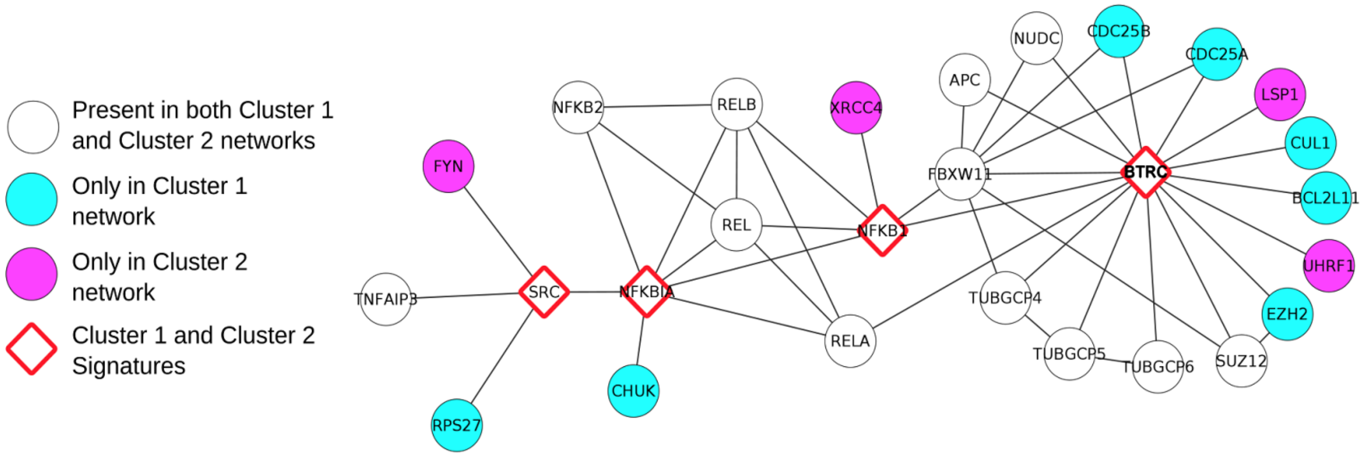

Supplementary Figure 9 – Interactors of *BTRC* gene in cluster 1 and cluster 2 signatures.

Supplementary Note 4

We tested our approach on a different PPI network, obtained as the logic union of the Bioplex-Ontocancro network with the intersection of the PPI networks in both Rolland *et al* (2014) [1] and Menche *et al* (2015) [2], obtained with different experimental techniques. Then, the CLR matrices of each tumor cluster correlation profile (see Methods) was superimposed to this network, and the Spectral Centrality (SC) measure was computed. We compared the new signatures (genes above the 90th percentile) with those proposed in the paper (Supplementary Table 4).

The new PPI network (before the CLR matrix superimposition) had the same number of nodes than Bioplex-Ontocancro original network, with 1045 links instead than 981 (6% more links). After CLR superimposition, the final number of added links in each cluster network was approximately 4%. The Supplementary Table 5 shows the comparison of different centrality measures (Spectral, Betweenness, and Strength) in original and new PPI clusters.

The concordance between old and new signature genes are: 16/25 (64%), 14/27 (51%), 10/17 (58%) for cluster 1, 2, and 3, respectively, showing a general agreement with previous results (Supplementary Table 4). In particular, in cluster 2 signature, genes related to DNA metabolism (*CETN2*, *FANCB*, *H2AFX*, *ERCC1*, *ERCC4*, *XPA*) and Ubiquitin-Proteasome System (*PSMB3*, *PSMC3*) were also retrieved. Moreover, the PLK1 gene, used as target in the validation experiments, was also found in the new signature.

Supplementary Table 4 - Signature genes for the modified PPI network.

**Cluster 1**	*MLF1IP, NFKB2, PPP2R5D, SRC, CUL1, RPS27, CENPC1, NFKBIA, RELB, ALOX5, MIS12, PPP2CB, REL, BTRC, NFKB1, PMF1*
**Cluster 2**	*CETN2, XPA, PSMB3, ERCC1, PSMC3, CENPC1, MLF1IP, TUBGCP5, DSN1, H2AFX, NFKBIA, IL6R, NEDD1, TNFRSF10B, RPA2, FANCB, NUP43, SRC, ERCC4, MIS12, PLK1*
**Cluster 3**	*CDC27, PRKAR2B, GMNN, FBXO5, MAPRE1, FBXW11*

Supplementary Table 5 - Spearman's rank correlation values for the centrality measures (Spectral Centrality SC, Betweenness Centrality BC, strength W) on the nodes for the 3 clusters of the modified PPI network. The results refer to the whole node list ("All") or only to the signatures, obtained as the top 10% of the ranked measures ("90th").

	**Cluster 1**	**Cluster 2**	**Cluster 3**
**All: SC vs BC**	0.69	0.70	0.67
**All: SC vs W**	0.30	0.41	0.33
**90th: SC vs BC**	-0.18	0.01	-0.08
**90th: SC vs W**	-0.38	-0.34	-0.29

Supplementary Note 5

To verify the robustness of our gene signatures to dataset perturbation (and avoid overfitting to the chosen dataset) we applied 100 repetitions, each one with 50% of patient random subsampling, of our analysis pipeline: 1) calculating the correlation matrix for all samples in one cluster; 2) applying Context Likelihood of Relatedness and selecting correlations with z>0 (see Methods); 3) obtaining cluster-specific networks; and 4) defining cluster gene signature as the first decile of the nodes sorted through Spectral Centrality measure. Then we counted how many times the original signature genes appeared in each subsampling procedure.
We found that most of the signature genes are conserved in these subsampling: 25/25 (100%) signature genes of cluster 1 were found at least one time after the patient subsampling, 23/27 (85%) in cluster 2; 17/17 (100%) in cluster 3 (see Supplementary Table 6, clusters 1-3 from left to right, with gene name and count of the appearances during subsampling), with about 30% of all genes appearing in at least 50% of the generated signatures for all three clusters.

Supplementary Table 6 - Percentage of repetitions that each gene resulted as signature when 50% of patient sub-sampling was performed before every run of the analysis pipeline.

**Cluster 1**		**Cluster 2**		**Cluster 3**	
**Gene**	**%**	**Gene**	**%**	**Gene**	**%**
ALOX5	73	*NEDD1*	99	*CDC27*	62
BTRC	73	*PSMC3*	97	*KIF2B*	61
PSMB9	69	*TUBGCP5*	97	*GMNN*	55
CUL1	64	*BTRC*	72	*FBXO5*	51
PSMF1	62	*SRC*	58	*FBXW11*	45
SRC	62	*NFKBIA*	49	*AKT2*	42
PMF1	60	*FANCB*	47	*E2F6*	41
NFKB2	55	*IL6R*	47	*PSMD9*	40
NFKBIA	54	*FYN*	45	*TFDP1*	37

CDC20	45	*CETN2*	44	*TNFRSF1A*	23
BUB1	43	*MIS12*	41	*TNFRSF10A*	21
RELB	43	*PSMB3*	38	*KIF2C*	20
NFKB1	41	*H2AFX*	36	*ANAPC1*	16
PSMC2	41	*TNFRSF10B*	34	*PSMC6*	14
RAD21	41	*NFKB1*	31	*PRKAR2B*	9
NDC80	33	*PARP1*	21	*CCNH*	5
MIS12	27	*RPA2*	20	*MAPRE1*	4
CENPC1	26	*DSN1*	15		
MLF1IP	24	*TUBGCP6*	9		
PPP2R5D	19	*ERCC4*	2		
RPS27	17	*ERCC1*	1		
STAG1	15	*XPA*	1		
PPP2CB	12	*PLK1*	1		
REL	12	*CENPC1*	0		
CHUK	7	*MCM10*	0		
		MLF1IP	0		
		NUP43	0		

Supplementary Note 6

We observed that the signature of the tumor cluster 2 (BRCA, GBM, LUAD, LUSC, OV, and UCEC) contains the *SRC*, *NFKB*, and *IL6R* genes, which participate to the activation of STAT3 transcription factor. We compared the expression level of STAT3 among clusters and observed that its expression was higher in tumors of the cluster 2 in comparison with the tumors of cluster 1 and 3 (one-way ANOVA p-value: 5.58×10^{-15}, Supplementary Figure 10).

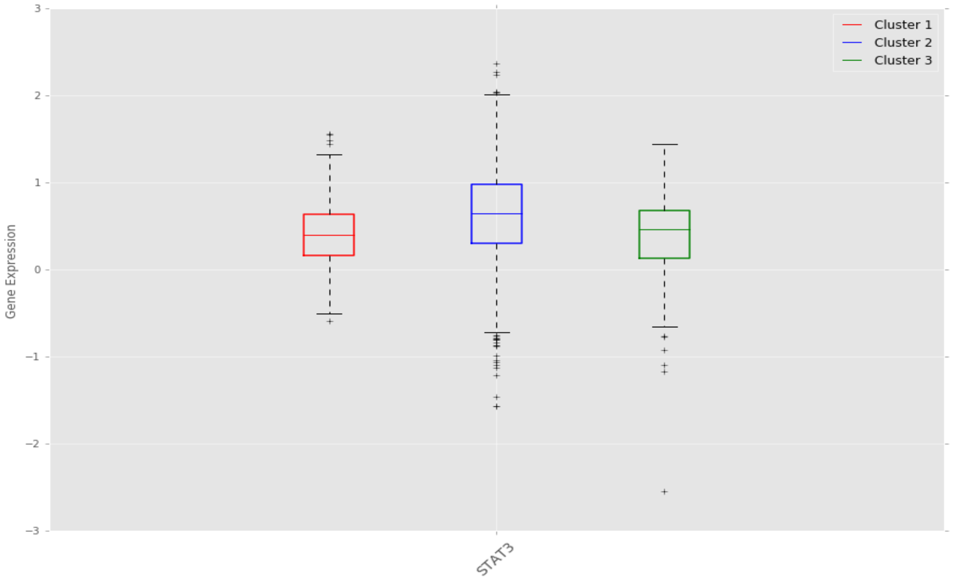

Supplementary Figure 10 – Cluster 2 patients show significantly higher *STAT3* gene expression in comparison with cluster 1 (two-sided Student's *t* test p-value: 1.08×10^{-9}) and cluster 3 (Student's *t* test p-value: 1.14×10^{-8}). The continuous horizontal line is the median, the lower and upper boundary represents the 25th and 75th percentiles, respectively. Whiskers extend to data points that lie within 1.5 Interquartile Ranges of the lower and upper quartiles; and observations that fall outside this range are displayed independently.

Supplementary Note 7

We evaluated the proximity of signature and mutated genes in the cluster networks. We observed no significant enrichment of mutated genes in the largest components, in comparison with the full network (two-sided Fisher's exact test p-values: 0.83, 0.86 and 1, for the cluster networks 1, 2 and 3, respectively). Moreover, we evaluated the overlap between the mutated-gene-lists and the signature-gene-lists, finding no significant intersection (Supplementary Table 7)

Supplementary Table 7 - Overlap between mutated genes and signature genes

	Number of Mutated Genes	Number of Genes in the Signature	Overlap: Mutated Genes in the Signature	Fisher exact test p-values (two-sided)
Cluster 1	16	25	2	0.5025
Cluster 2	27	27	2	0.7887
Cluster 3	14	24	1	0.7751

The complete list of mutated genes from Cosmic present in the Bioplex-Ontocancro network consists of 105 genes, with a minimal overlap with the signature-gene-lists: 3 genes in cluster 1 (*NFKB2, RAD21, REL*), 2 genes in cluster 2 (*ERCC4, XPA*) and 1 gene in cluster 3 (*AKT2*)

The relevance of our findings for drug targeting is better represented by the "proximity" of the gene signature to mutated genes, rather than a simple overlap between the two groups. To quantify the proximity of gene signatures to mutated genes we located the nearest mutation (in terms of shortest paths on the network) for each signature gene, resulting in a collection of minimal distance values for each cluster. The average minimal distance from the mutated genes $\langle d_{min} \rangle^{real}$ was then calculated for each cluster and tested with a permutation test. We performed 10^6 permutations of the signature labels and recalculated the average minimal distance. The p-values were calculated as:

$$p = \frac{\sum_{i=1}^{10^6} \langle d_{min} \rangle^i < \langle d_{min} \rangle^{real}}{10^6} \quad (1)$$

The results are reported in Supplementary Figure 11: signatures of cluster 1 and 2 are significantly closer to mutated genes than expected (random permutation test p-value = 9 x 10^{-4} and 6.9 x 10^{-3}, respectively). Signature of cluster 3 did not show significant proximity (p-value = 0.52).

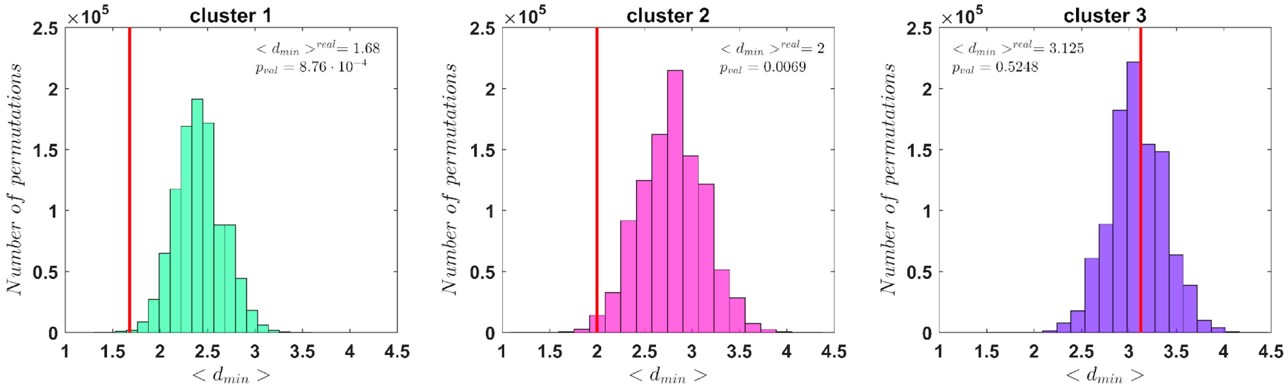

Supplementary Figure 11 - Distribution of the 10^6 permutations for the 3 clusters (from left to right). The insets show the average distances for the true signatures (represented in the plots as red vertical lines), and random permutation p-values with respect to the permutations.

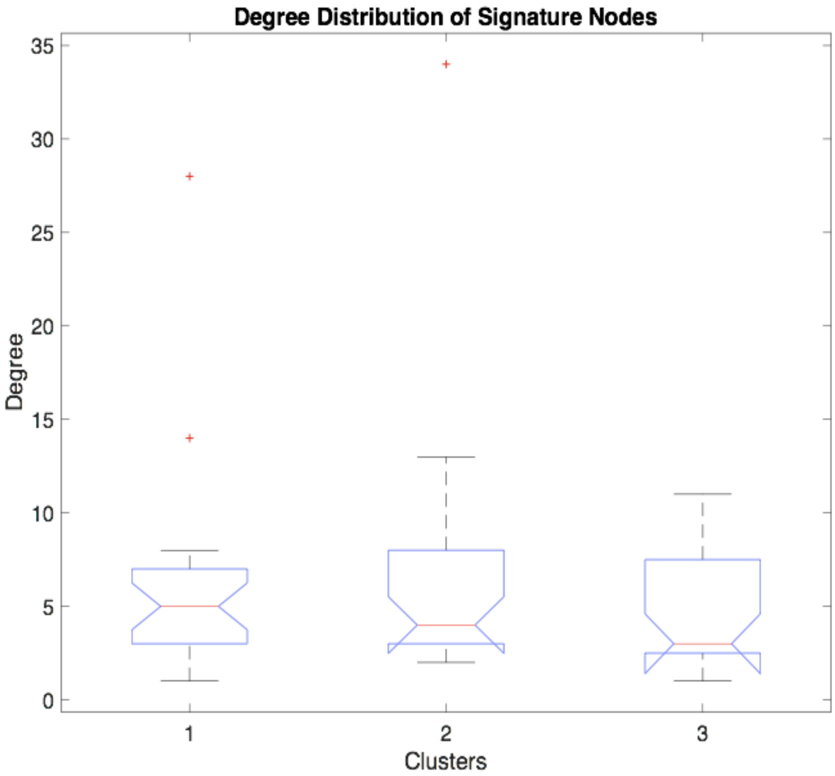

Supplementary Figure 12 - Degree distribution of the signature genes in the three clusters. The continuous horizontal line is the median, the lower and upper boundary represents the 25th and 75th percentiles, respectively. Whiskers extend to data points that lie within 1.5 Interquartile Ranges of the lower and upper quartiles; and observations that fall outside this range are displayed independently.

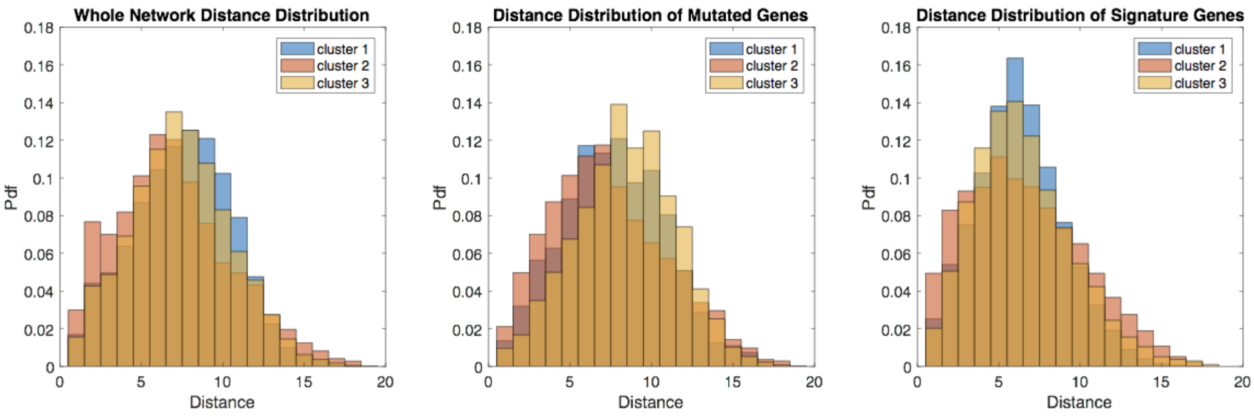

Supplementary Figure 13 - Distance Distributions for (from left to right): the whole networks, the Mutated Genes and the Signature Genes, respectively.

We further investigated the topological features of the three networks in several ways. First, we compared the degree distribution of the signature genes in each cluster network (See Supplementary Figure 12). The three distributions are similar, except for 3 outliers with higher degree: 2 nodes in Cluster 1 and 1 node in Cluster 2 (red asterisks). To evaluate the relevance of these outliers in the proximity tests, we ran the permutation tests a second time, removing these nodes from the signatures. The signatures of Cluster 1 and Cluster 2 were still significantly closer to mutated genes than expected (one-side permutation test p-values 0.0016 and 0.0027, respectively) while Cluster 3 remained not significant.
To understand the possible cause of the non-significant proximity in cluster 3, we analyzed the distribution of the distances between nodes in the three clusters (Supplementary Figure 13) but we did not observe any relevant difference in the three cases.
Additionally, we compared node distance distribution of mutated genes and signature genes with the nodes of the whole network, by computing Cohen's *d* effect size:

$$d_{\text{Cohen}} = \frac{\langle d_1 \rangle - \langle d_2 \rangle}{\sqrt{\frac{(n_1-1)s_1^2+(n_2-1)s_2^2}{n_1+n_2-2}}} \qquad (2)$$

where d_1 is the whole network distance distribution, and d_2 either the mutated-gene or the signature-gene distributions. Angle brackets stand for average value (Supplementary Table 8).

Supplementary Table 8 - *Cohen's d* effect size for Mutated Genes and Signature Genes

	Cluster 1	Cluster 2	Cluster 3
d_{Cohen} Total vs Mutated	-0.0555	-0.1187	-0.3226
d_{Cohen} Total vs Signature	0.4186	0.1120	0.3023

The negative values of the mutated-gene lists mean that they have higher average distance as compared to the whole network, while the signature-gene-lists show smaller average distances. Cluster 3 shows the biggest difference between these two values (0.62, versus 0.47 of cluster 1 and 0.23 of cluster 2), providing a possible explanation about why gene signatures were not significantly closer to mutated genes (Supplementary Figure 11): signature genes appear more in the center of the network while mutated genes are strongly peripheral, thus increasing the average minimal distance between the two groups.

Supplementary Note 8

We asked if drugs targeting signature genes are overrepresented in the ClinicalTrials.gov database, which would support our hypothesis that the applied method retrieves biological and clinically relevant targets for cancer treatment.
The information of drug-gene interactions was retrieved from the Drug Gene Interaction Database (DGIdb), considering only FDA approved drugs. We selected all studies in ClinicalTrials.gov that evaluated conditions related to cancer; and we discarded studies that involved conditions not only related to cancer but also to other diseases.
The comparison was performed using the Fisher's exact test. By considering all drugs that target genes present in the BioPlex-Ontocancro network, the enrichment was significant for drugs targeting genes in cluster 2 signature (two-sided Fisher Exact Test p-value = 0.0015) and in the border line of significance for those targeting genes in cluster 3 (two-sided Fisher's Exact Test p-value = 0.085, Supplementary Table 9). Due to the small number of samples, we remark that for cluster 3 just one more drug counted in the "Drug targeting signature genes in clinical trials" (i.e. 7 vs 123) would have led to a significant difference (Fisher's Exact Test p-value = 0.045). Anyway, the trend is what would be expected for all three clusters (at least a double odds ratio in the worst case of cluster 1).

Supplementary Table 9 - Enrichment in ClinicalTrials.gov of drugs targeting signature genes in relation to drugs targeting genes in the BioPlex or BioPlex-Ontocancro networks

		Drugs targeting genes in the BioPlex-Ontocancro Network		
		**Drugs Targeting Signature Genes**	**Drugs Targeting Non-signature Genes**	**Fisher's Exact Test P-value (two-sided)**
**Cluster 1**	in Clinical Trials	18	111	0.21
	not in Clinical Trials	20	195	
**Cluster 2**	in Clinical Trials	22	107	0.0015
	not in Clinical Trials	13	202	
**Cluster 3**	in Clinical Trials	6	123	0.085
	not in Clinical Trials	3	212	

Supplementary Note 9

For each cluster, we separated individuals in two classes according to the expression levels of the genes in the signature (through k-means clustering), and tested if they could predict patient survival outcome (Methods section in the main text). For cluster 1 and 3, survival information were available only for 17 and 32 patients, respectively, which resulted in non-significantly different survival curves (Supplementary figures 14 and 15).

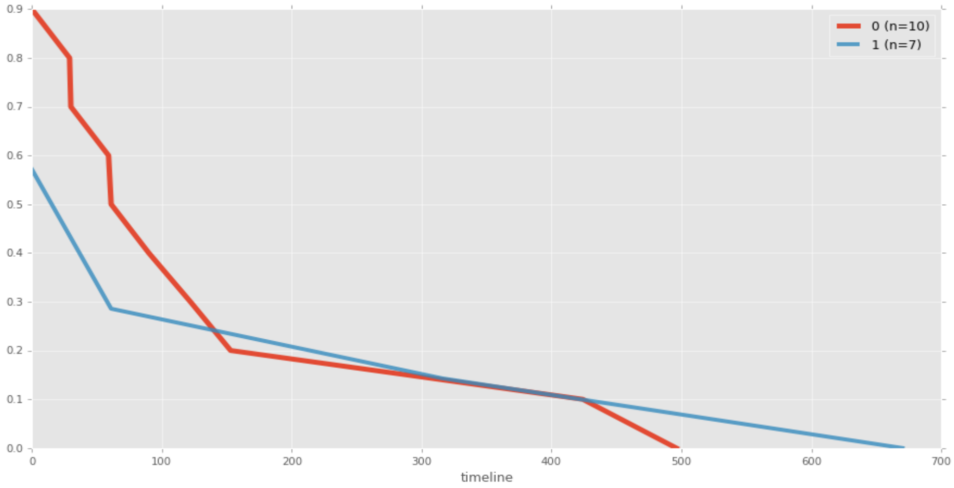

Supplementary Figure 14 - Kaplan-Meier curves for the two groups of cluster 1 patients. The clustering was applied considering only the genes in cluster 1 signature. Log rank-test p-value: 0.9118.

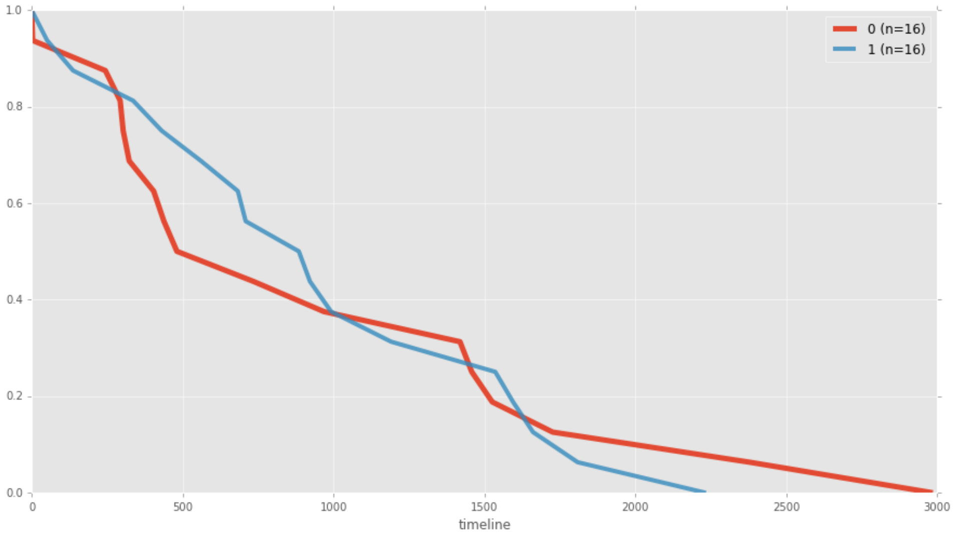

Supplementary Figure 15 - Kaplan-Meier curves for the two groups of cluster 3 patients. The clustering was applied considering only the genes in cluster 3 signature. Log rank-test p-value: 0.9056.

For cluster 2, the patients having survival information were 790 (out of 889) and 1094 (out of 1130) for K-means groups 0 and 1, respectively. The ratio of censored samples is shown in Supplementary Table 10.

Supplementary Table 10 - Percentage of censored samples for the tumour types in each k-means group.

	Total Censored (%)	Censored in Group 0 (%)	Censored in Group 1 (%)
**BRCA**	12.92	12.22	13.73
**GBM**	76.62	84.37	75.05
**LUAD**	12.90	21.05	0.00
**LUSC**	42.20	41.13	53.84
**OV**	51.83	50.71	58.53
**UCEC**	12.96	17.50	0.00

We observed that, for cluster 2, the signature significantly stratified individuals according to their survival outcome (Figure 3 main text). To evaluate the robustness of this result, we repeated the K-means clustering procedure and evaluated the log-rank test with 1000 random signatures (with same size as the original signature). For each random signature, we measured also S_T, the unbalancing of tumor samples division into the two groups:

$$S_T = < \left| \frac{T_0 - T_1}{T_0 + T_1} \right| > \quad (3)$$

being T_0 and T_1 the number of patients from each tumor assigned to the groups 0 and 1, respectively, and angle brackets represent average over the 6 tumours. The S_T values vary between 0 (patients equally distributed between groups) and 1 (all patients of one tumour assigned to one group).

Supplementary Figure 16 shows that the more unbalanced the clusters, the smaller the resulting p-values for the survival test. If we consider only random signatures which result in an average $S_T < 0.5$, the true signature outperforms 95% of the random signatures.

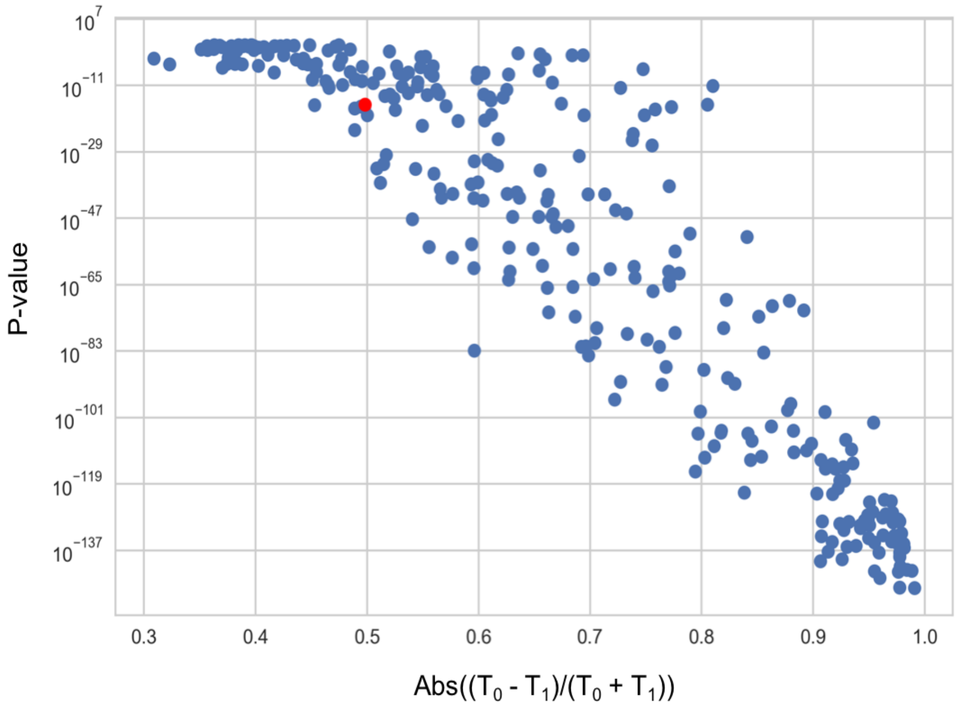

Supplementary Figure 16 - Log-rank test p-value (y axis) vs S_T score (x axis) produced by 1000 random signatures for cluster 2. The red dot represents the true signature.

We also performed a multivariate survival analysis considering the following covariates: age at initial pathologic diagnosis (10-94 years), tumor type (1-6), and k-means group (0,1). We checked the Proportional Hazard assumption of the Cox Model by applying the cox.zph (Survival R package). We observed that the Cox Proportional Hazard Model was not suitable for the data (p-value < 0.05). Therefore, we applied the Aalen's Additive model (P-values computed with the R package Survival, cumulative regression coefficients and cross-validation computed with the Python Lifelines package). 5-fold cross-validation reports a mean concordance of 0.67. We can see in Supplementary Table 11 that the effects for all covariates were significant, with grouping showing the largest effect.

Supplementary Table 11 - Regression coefficients and p-values for Aalen's Additive model

Covariate	Coefficient	P-value
Age	1.87×10^{-5}	6.93×10^{-11}
Tumor Type	1.91×10^{-4}	3.53×10^{-8}
K-means Group	-4.50×10^{-4}	2.88×10^{-8}

We investigated the association between survival time and the covariates: age, k-means clustering group, and pathologic stage (BRCA, LUAD, LUSC) or neoplasm histologic grade (UCEC, OV), considering each tumor individually. GBM did not present histologic or pathologic grading data and was not considered for this analysis. The k-means clustering group was calculated for each tumor separately, considering the expression profiles of genes present in the signature. For each tumor we checked the Proportional Hazard

assumption of the Cox Model. For the tumors in which the assumption held (LUAD, LUSC, UCEC) we applied the Cox Proportional Hazard Model and for the others (OV and BRCA) we applied the Aalen's Additive model.

The results are presented in Supplementary Table 12. The analysis showed a significant contribution of the gene signature for LUSC (154 samples, 8 different stages) and a borderline significance for OV (Aalen’s Additive Model p-value = 0.08). However, we remark that this further stratification significantly reduces the statistical power of the test applied. For two tumours (LUAD, UCEC) a small number of patients is further stratified, resulting in groups having at most 20 samples. The remaining tumours (BRCA, OV) have a large number of samples (>500) but they are distributed unevenly inside the tumour stage groups: in BRCA almost 85% of the samples are in one stage, while one significant stage contains only 1 sample.

Supplementary Note 10

In order to extend our conclusions of the experimental validation results to a larger set of cell lines and drugs, we compared our results with those reported in the Genomics of Drug Sensitivity in Cancer (GDSC) project [3]. The project reports the drug screening results for 224,510 drug-cell line pairs (265 drugs, 1074 cell lines).

First, we compared the IC50 values obtained in our *in vitro* experiments with: 1) those of all drug-cell pairs, and 2) those of drugs tested only in the cell lines MCF-7 and TG98 (217 and 216 drugs, respectively). In the first case, all pairs presented lowers IC50 values than the average: 0th, 6th, 10th, and 24th percentiles for the pairs Bortezomib-MCF7, Bortezomib-TG98, BI6727-MCF7 and BI6727-TG98, respectively (Supplementary Figure 17). With regards to all experiments performed specifically in the cell lines MCF-7 and TG98, Bortezomib and BI6727 presented lower IC50 values than the average in both cell models: 9th and 22nd percentile for MCF-7, 0th and 7th percentile in T98G cell line (Supplementary Figure 18).

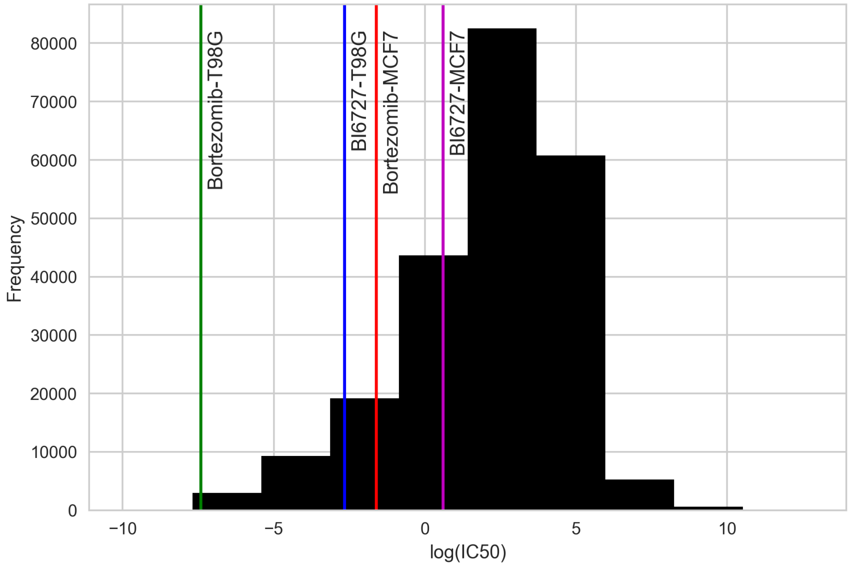

Supplementary Figure 17 - Distribution of log(IC50) values for all drug-cell line pairs in the GDSC project. The vertical lines point to the log(IC50) values observed in our experiments. Percentiles: 0th, 6th, 10th, and, 24th for the Bortezomib-TG98, BI6727-TG98, Bortezomib-MCF7 and BI6727-MCF7 pairs, respectively.

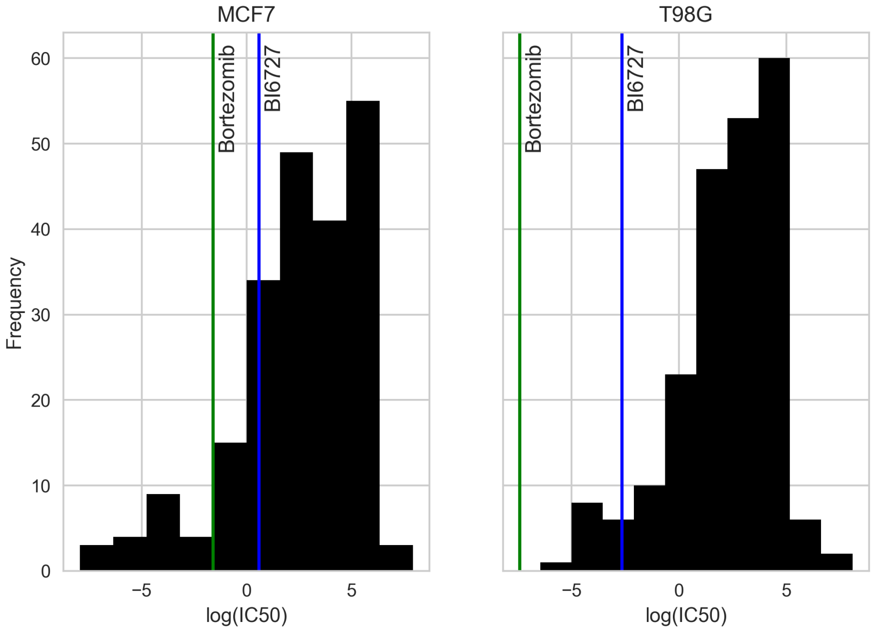

Supplementary Figure 18 - Distribution of the log(IC50) values for all drugs tested in the cell lines MCF7 and T98G. The vertical lines point to log(IC50) values observed in our experiments. Percentiles: 9th, 22th, 0th, and, 7th for the Bortezomib-MCF7, BI6727-MCF7, Bortezomib-TG98 and BI6727-TG98 pairs, respectively.

We manually mapped the cell lines in the GDSC project according to the clusters of tumors obtained in this study (Supplementary Table 26). By using drug-gene interactions from the DGIdb and GDSC, we observed that most cell lines from each cluster (51/53, 103/218, and 47/49, respectively) presented lower IC50 values when treated with drugs targeting genes in the signature than when treated with other drugs (Supplementary Figures 19-21). This trend was also observed when the comparison was made between cells treated with drugs targeting genes in the signature and drugs targeting non-signature genes in the BioPlex-Ontocancro network: 50/53, 63/218, and 40/49 cell lines, for which 2, 6, and 4 cell lines (clusters 1, 2, and 3, respectively) the difference was statistically significant (two-sided Student's *t* test, p-value <0.05, Supplementary Figure 22).

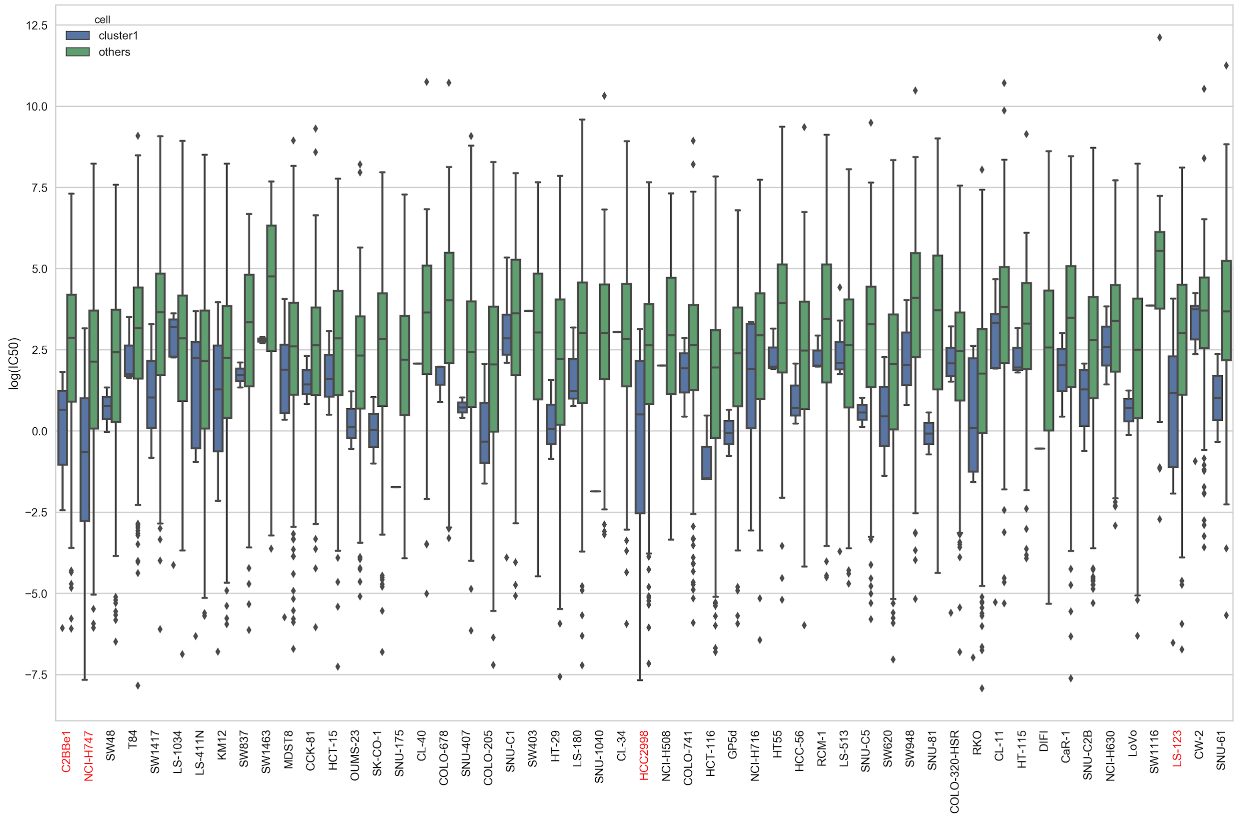

Supplementary Figure 19 - Boxplot showing the IC50 values of cell lines (n=53) that correspond to tumours in cluster 1: treated with drugs targeting genes in the signature (blue) and treated with all other drugs (green). Red labels show cell lines (n=4) in which the differences were statistically significant (two-sided Student's *t* test p-value < 0.05). The continuous horizontal line is the median, the lower and upper boundary represents the 25th and 75th percentiles, respectively. Whiskers extend to data points that lie within 1.5 Interquartile Ranges of the lower and upper quartiles; and observations that fall outside this range are displayed independently.

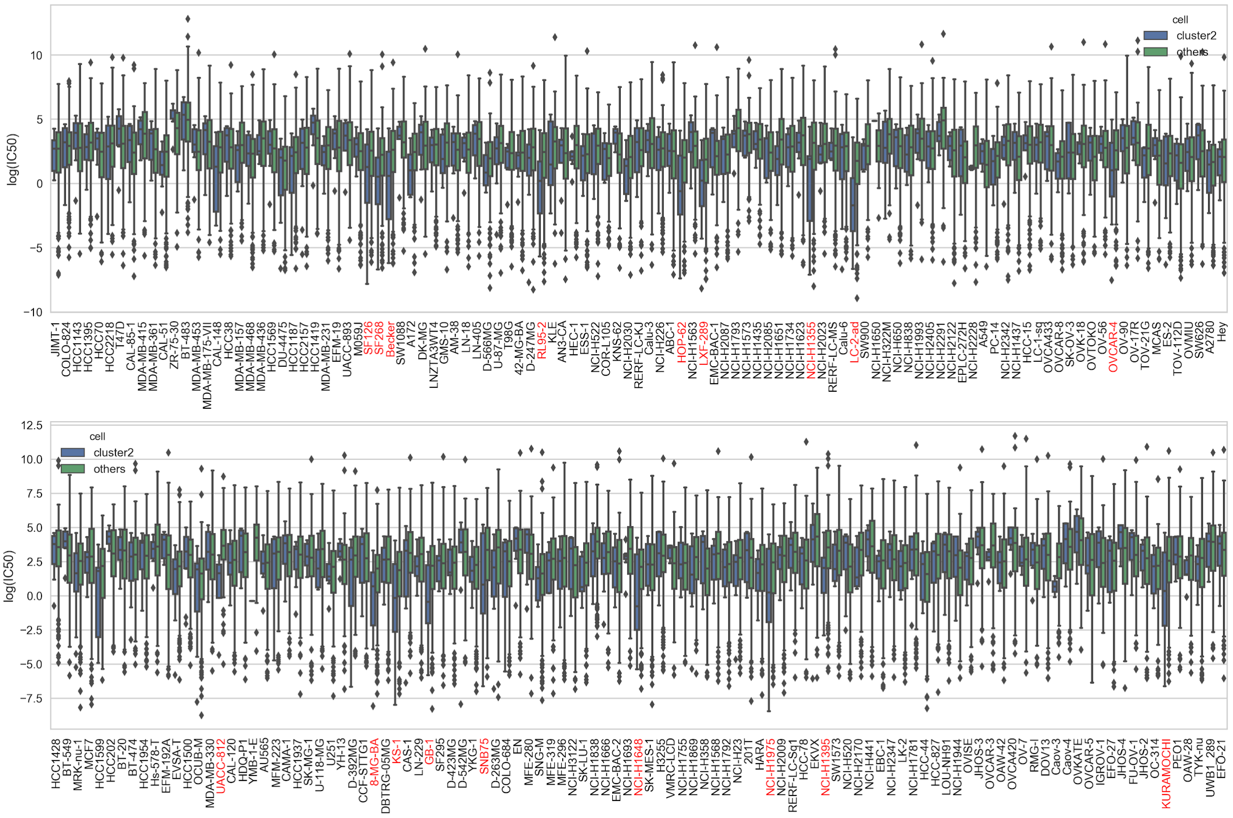

Supplementary Figure 20 - Boxplot showing the IC50 values of cell lines (n=218) that correspond to tumours in the cluster 2: treated with drugs targeting genes in the signature (blue) and treated with other drugs (green). Red labels show cell lines (n=18) in which the differences were statistically significant (two-sided Student's *t* test p-value < 0.05). The continuous horizontal line is the median, the lower and upper boundary represents the 25th and 75th percentiles, respectively. Whiskers extend to data points that lie within 1.5 Interquartile Ranges of the lower and upper quartiles; and observations that fall outside this range are displayed independently.

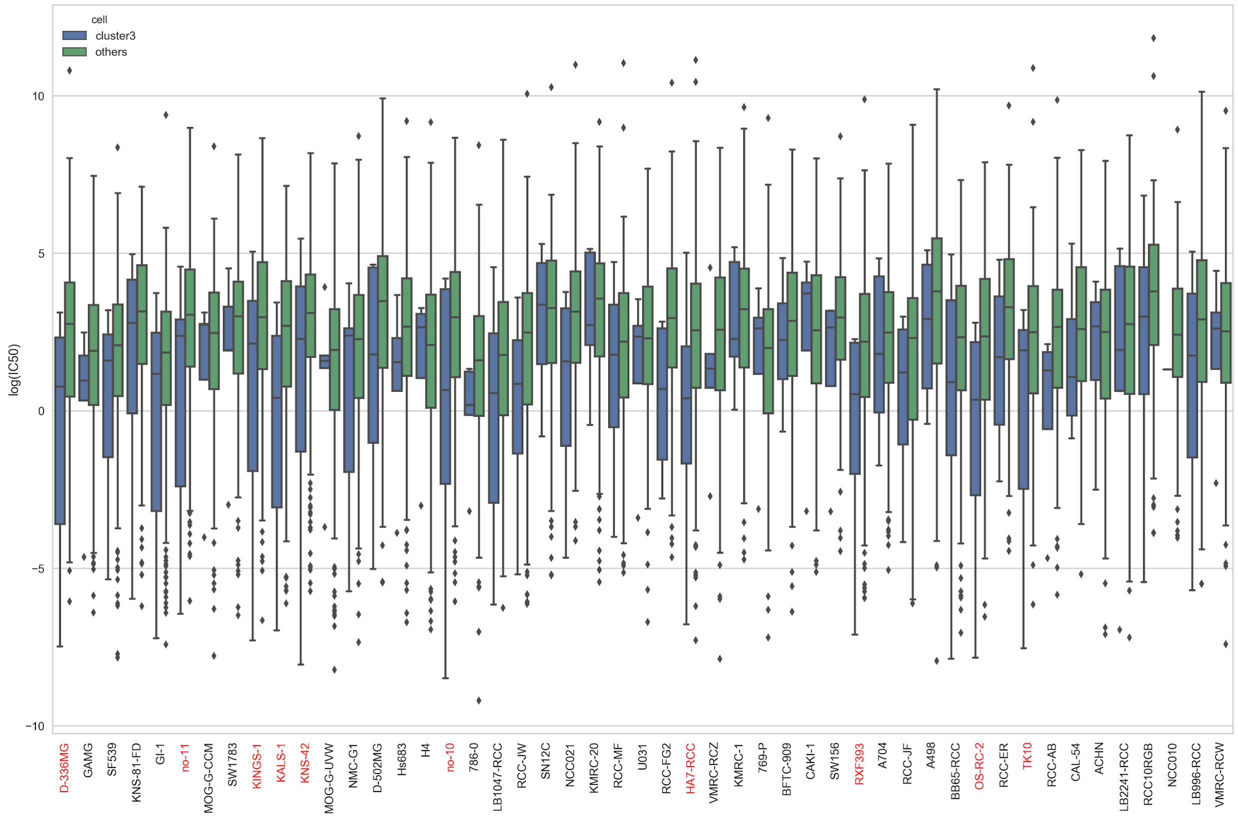

Supplementary Figure 21 - Boxplot showing the IC50 values of cell lines (n=49) that correspond to tumours in the cluster 3: treated with drugs targeting genes in the signature (blue) and treated with other drugs (green). Red labels show cell lines (n=10) in which the differences were statistically significant (two-sided Student's *t* test p-value < 0.05). The continuous horizontal line is the median, the lower and upper boundary represents the 25th and 75th percentiles, respectively. Whiskers extend to data points that lie within 1.5 Interquartile Ranges of the lower and upper quartiles; and observations that fall outside this range are displayed independently.

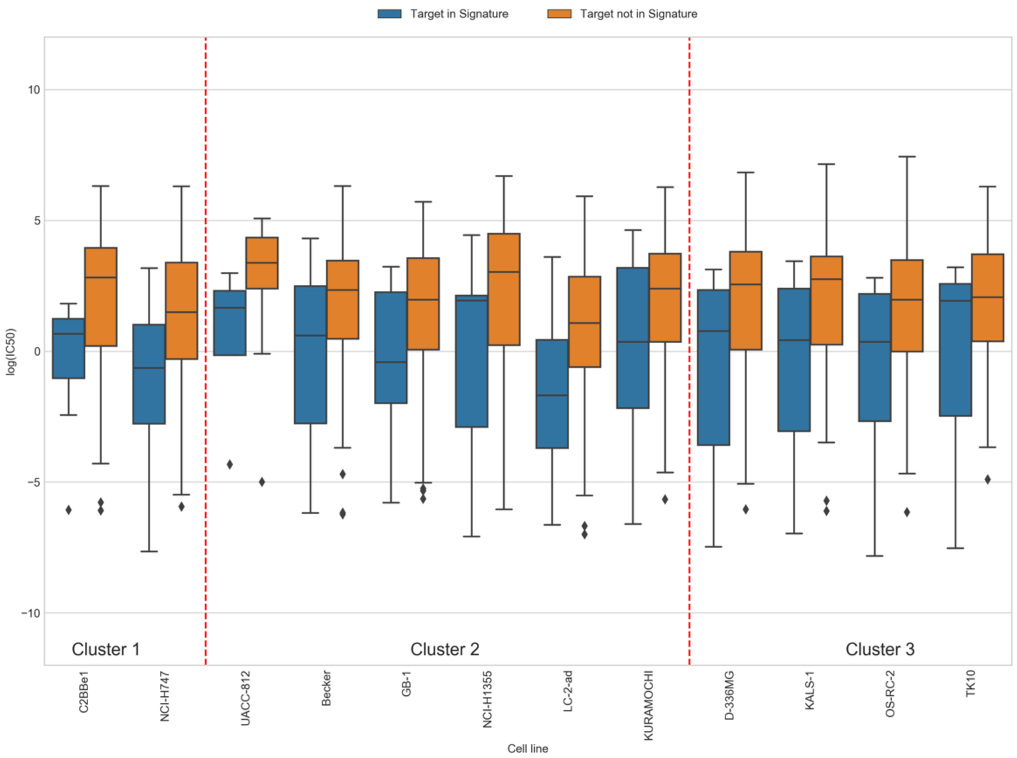

Supplementary Figure 22 - Cell lines that show significantly lower IC50 values (two-sided Student's *t* test p-value < 0.05) when treated with drugs targeting signature genes. We retrieved drug screening data from the GDSC project and considered the cell lines associated to the tumours in our clusters. The continuous horizontal line is the median, the lower and upper boundary represents the 25th and 75th percentiles, respectively. Whiskers extend to data points that lie within 1.5 Interquartile Ranges of the lower and upper quartiles; and observations that fall outside this range are displayed independently Blue boxplots: IC50 values for drugs targeting signature genes; orange boxplots: IC50 values for drugs targeting non-signature genes.

Supplementary Table 12 - Top decile genes measured by Spectral Centrality in the whole Bioplex-Ontocancro network

ALOX5	PIK3CA
APP	PIK3CB
C17orf70	PIK3CD
CCDC99	PIK3R2
CETN2	PIK3R3
CSNK2A1	PLK1
CSNK2A2	POLA1
EME1	POLA2
ERCC1	PRIM1
ERCC4	PRIM2
ERCC6L	PSMB3
FANCB	PSMC3
GAB1	RAC1
GRB2	SEC13
H2AFX	TNF
IL6R	TNFRSF1A
MAP4K5	TRAF6
MCM10	UBB
MLF1IP	UBE2T
MUS81	XPA
NFKBIA	XPC
NRP1	

Supplementary References

1. Rolland, T., Taşan, M., Charloteaux, B., Pevzner, S. J., Zhong, Q., *et al*. (2014). A proteome-scale map of the human interactome network. *Cell*, *159*(5), 1212–1226.
2. Menche, J., Sharma, A., Kitsak, M., Ghiassian, S. D., Vidal, M., Loscalzo, J., & Barabási, A.L. (2015). Uncovering disease-disease relationships through the incomplete interactome. *Science*, *347*(6224), 1257601.
3. Yang, W. *et al.* Genomics of Drug Sensitivity in Cancer (GDSC): a resource for therapeutic biomarker discovery in cancer cells. *Nucleic Acids Res.* **41,** D955-61 (2013).
