# Supplementary material for: Network integration of multi-tumour omics data suggests novel targeting strategies
Source: Nat Commun. 2018 Oct 30;9:4514. doi: 10.1038/s41467-018-06992-7 (PMC6207774; doi:10.1038/s41467-018-06992-7)
Supplement: Supplementary file 3 — Description of Additional Supplementary Files [file 41467_2018_6992_MOESM3_ESM.docx]

Supplementary Data 1 - Multivariate Survival Analysis performed separately in each tumor dataset.

Supplementary Data 2 - Gene Ontology Enrichment Analysis for the cluster 1, 2 and 3 gene signatures.

Supplementary Data 3 - Protein interactions out of the largest component of cluster 1,2 and 3 networks.

Supplementary Data 4 - List of drug-gene interactions for the signature genes, extracted from the Drug Gene Interaction database (DGIdb).

Supplementary Data 5 - List of ongoing clinical trials (according to ClinicalTrials.gov) that evaluate the inhibition of the signatures genes.

Supplementary Data 6 - Cluster 1, 2, and 3 gene signatures with the respective Spectral Centrality values and the drugs that target these genes according.

Supplementary Data 7 - Combination Indexes for BI6727 and Bortezomib treatment at different concentrations in both MCF-7 and MCF-7 cell lines.

Supplementary Data 8 - Cell lines present in the Genomics of Drug Sensitivity in Cancer (GDSC) project mapped to their respective tumour cluster.

Supplementary Data 9 - BioPlex-Ontocancro Network.

Supplementary Data 10 - Cluster 1 Network.

Supplementary Data 11 - Cluster 2 Network.

Supplementary Data 12 - Cluster 3 Network.
